# Effect of dialectical behaviour therapy on depression & anxiety among elderly individuals

**DOI:** 10.6026/973206300212352

**Published:** 2025-08-31

**Authors:** Ramani G., Tamizharasi K.

**Affiliations:** 1 Department of Psychiatric Nursing, Kongunadu College of Nursing, Coimbatore, The Tamil Nadu DR.M.G.R Medical University, Chennai, India; 2 Department of Paediatric Nursing, Sri Gokulam College of Nursing, Salem, The Tamil Nadu DR.M.G.R Medical University, Chennai, India

**Keywords:** Dialectical behavior therapy, depression, anxiety, elderly

## Abstract

Dialectical behavior therapy (DBT) is a therapeutic approach that can align a balance in a person's acceptance strategies with cognitive and behavioral change strategies. Therefore, it is of interest to evaluate the effectiveness of a 12-week Dialectical Behavior Therapy (DBT) program in reducing depression and anxiety among elderly individuals aged 65 years and above. Hence, a total of 40 participants were randomly assigned to either a DBT intervention group or a waitlist control group. Depression and anxiety levels were assessed before and after the intervention using the Geriatric Depression Scale (GDS) and the Hamilton Anxiety Rating Scale (HAM-A), respectively. Participants in the DBT group demonstrated significant reductions in both depression and anxiety scores compared to the control group (p < .05), with moderate to large effect sizes (Cohen's d = 0.72 for depression; d = 0.69 for anxiety). Thus, data shows that DBT is an effective intervention for alleviating emotional distress in elderly populations and may be beneficial for integration into community-based mental health services for aging individuals.

## Background:

Aging is frequently accompanied by increased vulnerability to emotional disorders such as depression and anxiety, driven by factors including declining physical health, loss of social roles, bereavement, and isolation [1]. These conditions significantly impact quality of life and functional independence among elderly individuals, yet they are often underdiagnosed and undertreated in this population [2]. Addressing emotional distress in older adults is therefore a pressing public health concern. Dialectical Behavior Therapy (DBT), originally developed for borderline personality disorder, incorporates mindfulness, emotion regulation, distress tolerance, and interpersonal effectiveness strategies [3]. While DBT's effectiveness is well-established in younger populations and individuals with mood disorders, recent research has extended its application to older adults, showing promise in improving psychological outcomes, including reductions in depressive and anxious symptomatology [4, 5]. Despite its potential, rigorous research evaluating DBT's effects on depression and anxiety specifically in elderly populations remains limited. Geriatric depression and anxiety often present differently than in younger individuals, making tailored interventions particularly valuable [6]. Standard pharmacological treatments are frequently complicated by comorbidities or polypharmacy in this age group, highlighting the importance of effective, non-pharmacological therapies [7]. Therefore, it is of interest to evaluate the effect of a 12-week DBT intervention on levels of depression and anxiety among elderly individuals aged 65 and above. Depression and anxiety were measured using the Geriatric Depression Scale (GDS) and the Hamilton Anxiety Rating Scale (HAM-A), respectively-both widely validated tools in geriatric populations [8, 9]. We hypothesize that participants receiving DBT will exhibit significantly greater reductions in depressive and anxiety symptoms compared to those in a waitlist control group, thereby contributing valuable evidence for the integration of DBT into mental health care for the elderly [10, 11]. Therefore, it is of interest to evaluate the effect of dialectical behaviour therapy on depression among elderly individuals.

## Methodology:

## Study design and participants:

A randomized controlled trial was conducted among 40 elderly individuals aged 65 years and above (mean age 71.8 ± 4.9 years), recruited from community centers in City X. Participants were randomly assigned to either a DBT intervention group (n = 20) or a waitlist control group (n = 20).

## Inclusion and exclusion criteria:

Participants with mild to moderate emotional distress (GDS scores 5-10 and HAM-A scores 14-25) were included. Those with severe cognitive impairment (MMSE < 24), active suicidal ideation, or ongoing psychiatric treatment were excluded.

## Intervention:

The DBT group received a 12-week intervention with weekly 90-minute group sessions covering mindfulness, emotion regulation, distress tolerance, and interpersonal effectiveness. The control group received no treatment during the study period.

## Outcome measures:

Depression was assessed using the Geriatric Depression Scale (GDS), and anxiety was measured using the Hamilton Anxiety Rating Scale (HAM-A). Both tools were administered at baseline and post-intervention.

## Procedure and ethics:

Participants were screened, consented, and randomized using a computer-generated sequence. Post-assessments were conducted one week after the final session. Ethical approval was obtained from the University IRB, and participant confidentiality was maintained.

## Data analysis:

Data were analyzed using SPSS version 27. Paired-sample t-tests assessed within-group changes, independent-sample t-tests compared between-group outcomes and Cohen's d was used for effect size estimation. Significance was set at p < 0.05.

## Results:

Table 1 (see PDF) presents the demographic characteristics of participants, indicating that 55% of the DBT group and 60% of the control group were female, 60% and 55% respectively had education up to high school, and 70% in the DBT group and 65% in the control group were married. Most participants lived with family (80% DBT; 75% control), and the mean age was comparable (71.5 ± 4.7 years in DBT vs. 72.1 ± 5.1 years in control), confirming baseline demographic similarity. Table 2 and Figure 1 (see PDF) show that in the DBT group, mean GDS scores significantly decreased from 8.1 ± 1.2 to 5.4 ± 1.1, and HAM-A scores from 20.3 ± 2.8 to 14.7 ± 2.4 (p < 0.001 for both), with effect sizes of 0.72 and 0.69 respectively. In contrast, the control group showed negligible changes in GDS (8.0 ± 1.3 to 7.8 ± 1.4) and HAM-A (20.1 ± 2.9 to 19.6 ± 3.1), which were not statistically significant. These results, illustrated clearly in Figure 1 (see PDF), highlight the significant psychological benefit of DBT in reducing depression and anxiety among elderly participants.

## Discussion:

The findings of this randomized controlled trial demonstrate that a 12-week Dialectical Behavior Therapy (DBT) intervention significantly reduced symptoms of depression and anxiety among elderly individuals, as measured by the Geriatric Depression Scale (GDS) and the Hamilton Anxiety Rating Scale (HAM-A). Participants in the DBT group exhibited substantial improvements in emotional well-being, while the control group showed no significant changes over the same period. These results are consistent with prior studies suggesting that DBT's structured, skill-based approach is effective in managing affective symptoms in older adults [3, 4 and 5]. The observed reductions in both depression and anxiety scores align with the core principles of DBT, which emphasize mindfulness, emotion regulation, distress tolerance, and interpersonal effectiveness. These therapeutic components may be particularly beneficial in later life, when individuals often face psychological stressors such as social loss, chronic illness, or diminished autonomy [1, 2]. The effect sizes observed in this study (Cohen's d = 0.72 for depression and 0.69 for anxiety) indicate moderate to large clinical impact, supporting the hypothesis that DBT can serve as an effective non-pharmacological intervention in geriatric populations. This study builds on previous findings that DBT can reduce depressive symptoms in elderly individuals with comorbid personality disorders [4], extending the evidence base to include general older adults with subclinical emotional distress. While existing literature has primarily focused on emotion regulation outcomes or general psychological functioning, this trial contributes specific data on depression and anxiety as standalone outcome measures, reinforcing the versatility of DBT across symptom domains [5, 8]. The mechanisms underlying DBT's efficacy may involve enhanced self-awareness through mindfulness, improved emotional control, and reduced maladaptive coping-all of which are known to buffer against depression and anxiety in later life [3, 12]. Moreover, interpersonal effectiveness training may combat social isolation and perceived helplessness, both of which are recognized contributors to late-life psychological disorders [13]. However, several limitations must be acknowledged. The relatively small sample size (n = 40) and the single-center urban setting may limit generalizability, especially across culturally diverse populations [14]. The use of a waitlist control group, while ethically justified, may not control for non-specific therapeutic factors such as social interaction or attention from facilitators [15]. Additionally, the study only measured short-term outcomes; the sustainability of treatment effects remains to be evaluated. Despite these limitations, the results carry significant implications for community-based mental health strategies targeting older adults. DBT could be integrated into senior wellness programs, outpatient mental health clinics, or delivered via telehealth to reach homebound elderly individuals [8, 16]. Lynch *et al.* recommend that DBT skills training and telephone coach may offer promise to successfully augment the effects of antidepressant medication in depressed older adults [17]. Future studies should explore long-term follow-up, cost-effectiveness, and comparisons with other therapeutic modalities such as Cognitive Behavioral Therapy (CBT) [10].

## Conclusion:

Data support for the use of DBT as an effective intervention to reduce depression and anxiety in elderly individuals. Given the rising burden of mental health issues in aging populations, scalable and skill-based interventions like DBT hold considerable promise for enhancing emotional well-being and promoting successful aging.
